# AGREE-YOLO: A Framework for Seafood Recognition and Cross-Cultural Gastronomic Recommendation

**DOI:** 10.3390/foods15101795

**Published:** 2026-05-19

**Authors:** Mingxin Hou, Shucheng Liu, Jianhua Wei, Kunfang Zhi, Mingxin Liu, Cong Lin

**Affiliations:** 1 School of Mechanical Engineering, Guangdong Ocean University, Zhanjiang 524088, China; 2 College of Food Science and Technology, Guangdong Ocean University, Zhanjiang 524088, China; 3 The 75852 Troop of the Chinese People’s Liberation Army, Guangzhou 510062, China; 4 School of Electronics and Information Engineering, Guangdong Ocean University, Zhanjiang 524088, China; 5 Guangdong Provincial Key Laboratory of Intelligent Equipment for South China Sea Marine Ranching, Guangdong Ocean University, Zhanjiang 524088, China

**Keywords:** agent-based systems, seafood recognition, vision language models, deep learning, food style

## Abstract

Real-time visual recognition systems integrated with culturally adaptive reasoning are urgently demanded in globalized culinary scenarios. An agent-oriented framework, Agent-based Gastronomy Recommender Enhanced Engine with YOLO (AGREE-YOLO), is proposed in this study, which integrates an optimized lightweight YOLOv13 detector and vision language model (VLM)-driven agents for cross-cultural seafood recipe recommendation. The improved YOLOv13 is equipped with group shuffle convolution (GSConv) modules and Wise-IoU (WIoU) loss, which is validated on a refined underwater seafood dataset targeting sea cucumbers, sea urchins and scallops. It achieves 91.2% precision and 87.3% recall, with 3.9% and 4.2% increments over the baseline model, and maintains 2.0 ms inference speed. Detection outputs are structured and stored in a MySQL database, and a novel ChatFlow pipeline is constructed in the Dify platform to support natural language database querying. VLM-powered agents retrieve structured data and generate culturally tailored recipes and dish images automatically. Operational validation verifies that the end-to-end pipeline realizes seamless conversion from seafood images to personalized cross-cultural recommendations. This work provides an integrated solution for intelligent, culturally adaptive gastronomy in food informatics.

## 1. Introduction

Culinary traditions are becoming increasingly globalized. Consumer demand for personalized cross-cultural gastronomic experiences is also rising. As a result, intelligent seafood recommendation systems have emerged as a pivotal research frontier within agricultural and food technology innovation [1]. The global seafood industry, which is integral to nutrition, economy, and cultural heritage, faces growing demands for intelligent systems that can improve culinary experiences using technology [2,3,4]. Traditional gastronomic recommendation systems rely primarily on collaborative filtering, content-based text analysis, or simple image tagging and often lack the ability to interpret raw visual inputs or adapt recommendations to diverse cultural contexts and psychological preferences [5]. Recent advancements in deep learning offer promising pathways for bridging computer vision with contextual, agent-driven reasoning. Object detection and large language models (LLMs) are particularly instrumental in enabling more personalized and culturally resonant food guidance [6,7].

In this study, the agent-based gastronomy recommender enhanced engine with YOLO (AGREE-YOLO) is proposed. It is a novel, agent-oriented framework designed to connect YOLO-based seafood recognition with VLM-driven agents for cross-cultural gastronomic recommendation. The framework incorporates an optimized lightweight YOLOv13 model that is enhanced with group shuffle convolution (GSCov) modules [8,9] and Wise-IoU (WIoU) loss [10,11] to accurately detect three representative seafood ingredients—holothurians (sea cucumbers), echini (sea urchins), and scallops—from underwater imagery. Detection results are systematically stored in a structured My Structured Query Language (MySQL) database, thereby ensuring efficient management and retrieval.

The primary objective of this work is to develop an integrated system capable of bridging real-time seafood recognition with culturally adaptive gastronomic recommendation. To achieve this, a key focus has been placed on the design and implementation of the AGREE-YOLO framework’s ChatFlow pipeline within the Dify environment. This pipeline serves as a crucial intermediary, facilitating seamless interaction between the structured data stored in a relational database and the VLM-powered agents. By doing so, it enables intelligent agents to dynamically access YOLO detection results for subsequent analysis and the generation of personalized, culturally contextualized recipe recommendations. AGREE-YOLO is the first architecture, to the best of current knowledge, to integrate YOLO-based detection outputs into a relational database structure, enabling efficient data retrieval and real-time, culturally sensitive recipe generation.

Furthermore, conventional frameworks for multimodal food recommendation are rarely constructed on dedicated agent-oriented development platforms, which limits the flexible orchestration of multiple functional modules and iterative calls to large language models (LLMs). Dify, as a professional platform for agent construction and workflow customization, is adopted in this study. On this platform, sequential and conditional invocations of LLMs can be realized, and complex task flows for visual interpretation, data querying, and content generation can be established. Therefore, an agent-oriented recommendation system supported by structured visual detection and managed database access is developed to fill the gap in end-to-end, culturally adaptive multimodal gastronomic systems.

The remainder of this paper is structured as outlined below. Related research advances are summarized and analyzed in Section 2. The methodology, including system architecture, YOLO-driven seafood detection, database management, and agent-oriented workflow design, is elaborated comprehensively in Section 3. Experimental assessments of the detection model and operational validation of the AGREE framework are detailed in Section 4. The key findings, practical implications, and existing limitations are interpreted and discussed in Section 5. Finally, the work is concluded, and directions for future investigations are proposed in Section 6.

## 2. Related Work

In the domain of seafood, accurate visual recognition is particularly challenging because of morphological diversity, variable underwater imaging conditions, and the frequent occurrence of occluded or blurred targets [12]. You Only Look Once (YOLO) series models have demonstrated state-of-the-art performance in real-time object detection [13,14]. However, their application in end-to-end gastronomic systems remains underexplored, particularly for those requiring cross-cultural adaptation. Current frameworks often treat ingredient detection and recipe recommendation as isolated modules [15,16,17], thus neglecting the seamless, structured flow of visual data into culturally and psychologically informed culinary reasoning [18,19,20]. Furthermore, existing LLM-based food recommendation systems predominantly process textual inputs and fail to incorporate visual ingredient identification, which limits their applicability in real-world scenarios in which users interact with physical seafood products [21,22,23].

To address these research gaps, an integrated approach that combines robust visual detection with LLM-powered, agent-based reasoning is needed. Such a system must not only identify seafood ingredients accurately but also translate these detections into structured data, retrieve them intelligently, and generate recommendations that are culturally adaptive and psychologically satisfying. Recent works have begun exploring multimodal food analysis [24,25,26] and agent-based personalization [27,28]; however, a cohesive pipeline that unifies high-precision seafood detection, structured database intermediation, and cross-cultural LLM agent reasoning has not been established.

Critical analysis of recent advances reveals several methodological trends and persistent limitations. In the domain of underwater seafood detection, significant progress has been made in model architecture optimization. For instance, an improved YOLOv7 tiny framework has been proposed for sea treasure detection in complex underwater environments, demonstrating that multi-scale feature fusion and enhanced bounding box regression can substantially improve detection accuracy under challenging imaging conditions [29]. Similarly, MOA-YOLO, a YOLOv10-based algorithm, has been developed specifically for deep sea fish detection, addressing the challenges posed by small, dynamic targets in degraded underwater environments through architectural refinements that balance accuracy and computational efficiency [30]. These studies collectively indicate that lightweight, real-time detection is achievable for underwater seafood targets-a prerequisite for practical gastronomic applications-yet none of these works extend their detection outputs to downstream recommendation or reasoning tasks.

Parallel advancements have been made in LLM-driven food recommendation and recipe generation, though visual integration remains conspicuously absent [31]. The KERL (Knowledge-Enhanced Recipe Recommendation using Large Language Models) system represents a notable contribution. It integrates food knowledge graphs with LLMs. This integration provides personalized recipe recommendations and generates nutritional information. Given a natural language question, KERL first extracts entities and retrieves relevant subgraphs from a knowledge graph. This contextual information is then fed into an LLM to select recipes that satisfy user constraints. Although this approach highlights the power of knowledge-augmented LLM reasoning for food applications, it is restricted to textual inputs and knowledge graph retrieval. No capability for processing visual ingredient data is present. This limitation is critical in real-world scenarios where users may interact with physical seafood products or captured images rather than articulated textual queries.

Multi-agent frameworks have recently emerged for food and nutrition applications. A closed-loop LLM-driven multi-agent system was proposed for personalized meal planning, using vision, dialogue, and state management agents to estimate nutrients from food photos and adapt meal plans to user preferences [32]. This work demonstrates the feasibility of agent orchestration for food-related tasks, yet its focus remains on nutrition management and dietary logging rather than cross-cultural gastronomic recommendation. Likewise, the Food4All framework has introduced a multi-agent approach for real-time free food discovery, integrating heterogeneous data aggregation, lightweight reinforcement learning for location optimization, and online feedback loops. While innovative in its application domain, this framework does not incorporate visual ingredient recognition or cultural adaptation mechanisms.

A critical gap across all these recent advances is the absence of an end-to-end pipeline that unifies visual detection, structured data management, and agent-based cross-cultural reasoning. While YOLO-based detectors have achieved remarkable accuracy for underwater seafood targets, and LLM-driven agents have demonstrated powerful reasoning capabilities for food recommendation, no existing framework provides seamless integration from raw seafood imagery to personalized, culturally adaptive gastronomic recommendations through a structured intermediary database.

The AGREE-YOLO framework bridges this gap with a ChatFlow pipeline in Dify, linking YOLO detection outputs in a MySQL database to VLM-powered agents for natural language generation. Visual detection and agent-based reasoning are integrated with structured data intermediation. This unified approach departs from the isolated module designs of prior work and provides a functionally complete solution for intelligent gastronomy.

## 3. Methodology

### 3.1. System Architecture of AGREE-YOLO

**Figure 1:**
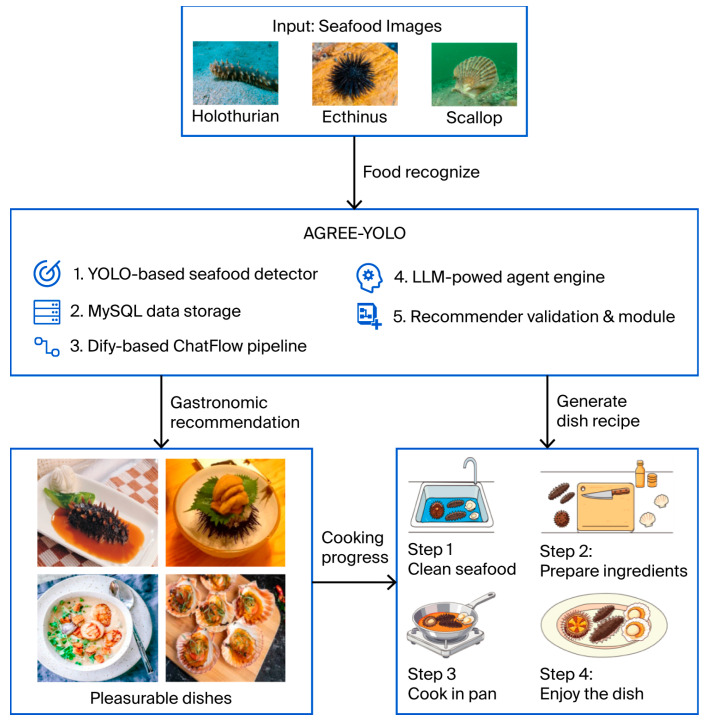
Overall system architecture of AGREE-YOLO.

The overall system architecture of AGREE-YOLO is illustrated in Figure 1. Computer vision-based seafood recognition is combined with agent-driven reasoning for culturally adaptive gastronomy recommendations. The architecture is composed of five interdependent modules. First, a YOLO-based detection unit processes raw image inputs and identifies representative seafood ingredients, including sea cucumber, sea urchin, and scallop. The detection outputs are then structured and stored within a MySQL 8.0.42.0 database, thereby ensuring efficient retrieval and traceability. Second, a ChatFlow pipeline developed in the Dify 1.4.2 environment establishes a communication layer between the database and the agent-based reasoning system. This pipeline facilitates seamless data exchange and provides contextualized ingredient information to the subsequent module. Third, a VLM-powered agent framework interprets the retrieved ingredient data and dynamically generates recipes that reflect diverse cultural practices while incorporating psychological dimensions of culinary satisfaction. Finally, the recommendations are delivered to the user via an adaptive interface designed to emphasize personalization and cultural relevance. By integrating visual recognition, structured data management, and agent-mediated reasoning, AGREE-YOLO operationalizes a comprehensive pathway from raw seafood imagery to cross-cultural gastronomic recommendations.

In the proposed AGREE-YOLO framework, the integration of Vision Language Models (VLMs) and Large Language Models (LLMs) plays a pivotal role in enabling culturally adaptive reasoning and personalized recipe generation. VLMs are leveraged to interpret and contextualize the visual information extracted by the YOLO-based detector, transforming raw image data into structured semantic representations. This process is facilitated by the VLM’s ability to understand and generate natural language descriptions based on visual inputs, thereby bridging the gap between computer vision and linguistic processing. Meanwhile, LLMs are employed to drive the agent-based reasoning system, utilizing their advanced natural language understanding capabilities to generate culturally tailored recipes and engaging cooking instructions. The synergy between VLMs and LLMs ensures that the framework can adapt to diverse culinary contexts and user preferences, providing a seamless and personalized gastronomic experience.

To illustrate the core logic of the proposed framework, a simplified pseudocode representation of the end-to-end workflow is presented in Algorithm 1.
**Algorithm 1** End-to-end workflow of AGREE-YOLO**Input:** Raw seafood image I, user natural-language query Q **Output:** Culturally adapted recipe R, dish image D1:   B ← YOLOv13_GSConv_WIoU (I)           → Bounding boxes & class labels 2:   **for** each detection b in B do3:          confidence ← b.confidence 4:          **if** confidence ≥ 0.83 **then**5:      store_into_MySQL (b.class_label, b.confidence, timestamp) 6:          **end if** 7:   **end for** 8:   refined_query ← USRR (Q)            → User Seafood Requirement Refinemen 9:   SQL_stmt ← rookie_text2data (refined_query)       → Convert NL to SQL 10: records ← rookie_execute_sql (SQL_stmt)     → Fetch from MySQ 11: cuisine ← CCS (records)              → Conditional Country Selection 12: R ← CMG (records, cuisine)            → Cooking Method Generato 13: D ← SDP (records, cuisine)           → Seafood Dish Picture Generator 14:   **return** (R, D)

### 3.2. YOLO-Based Seafood Detection

#### 3.2.1. Dataset Preparation

An experimental evaluation of the proposed seafood detection model was conducted using a refined subset of the Detecting Underwater Objects (DUO) dataset [33], which is a specialized computer vision benchmark that addresses challenges of dense and blurred targets in underwater scenarios. To ensure data integrity and reduce redundancy, the original DUO dataset underwent de-duplication via a perceptual hash algorithm, which yielded 6671 training images, 1111 testing images, and 1111 validation images with minimal interimage similarity. Drawing on culinary relevance, three representative seafood ingredients—holothurians, echini, and scallops—were selected as the target categories for this study, which corresponded to 7887, 50,156, and 1924 annotated instances, respectively.

To address the potential issue of dataset imbalance, which could adversely affect model performance, a stratified sampling strategy was employed during the training phase. This approach ensured that each seafood category was represented proportionally across training batches, thereby mitigating the impact of class imbalance. Furthermore, the model’s performance was evaluated using per-class metrics, including precision, recall, and F1-score, which provided detailed insights into its ability to recognize each seafood species accurately. These per-class results are reported in Section 4.1, offering a comprehensive assessment of the model’s performance across different categories.

**Figure 2:**
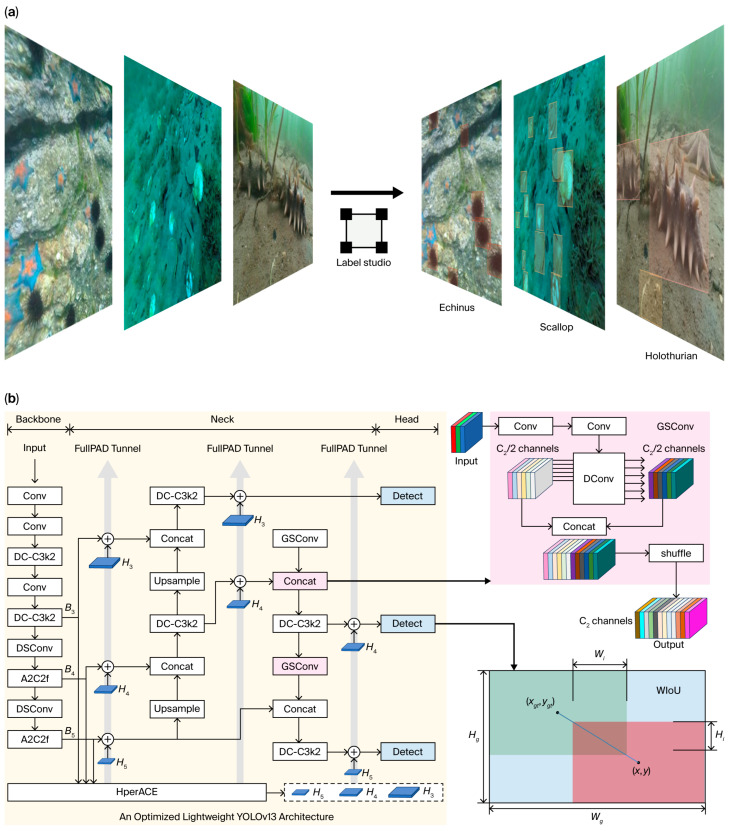
Seafood detection annotation and model architecture. (**a**) Annotation workflow for target seafood categories used in model training. (**b**) Structural design of the lightweight YOLOv13 model integrated with GSConv and WIoU loss for enhanced seafood detection.

As shown in Figure 2a, all the image annotations were performed using Label Studio 1.21.0, which is an open-source tool that supports precise bounding box labeling for object detection tasks. Notably, the original JSON-formatted tags of the DUO dataset were converted to TXT files to align with the input requirements of YOLO-based detection frameworks, thereby facilitating seamless integration into the training pipeline.

This dataset configuration retains the underwater environmental characteristics of the parent DUO dataset while emphasizing the practical gastronomic value of the selected ingredients. This approach supports culturally adaptive recipe generation. Potential dataset limitations are recognized, including restricted geographic representation and uneven sample distributions across the three target seafood categories. Inherent imaging biases associated with uniform underwater lighting and fixed capture angles are acknowledged as factors that may reduce cross-environment generalization.

#### 3.2.2. Lightweight, Real-Time-Optimized YOLOv13 Architecture

Architectural enhancements were made to the YOLOv13 model (Figure 2b), with the goal of achieving a lightweight and real-time-optimized object detection framework. Two key modifications are highlighted: the replacement of conventional convolutional (Conv) layers with GSConv modules and the integration of WIoU as the bounding box regression loss function.

The GSConv module, which applies a hybrid convolutional approach, is used to reduce the parameter count of the model while preserving the feature extraction efficacy. As depicted in Figure 2b, GSConv replaces standard Conv layers at two critical junctures within the YOLOv13 architecture. This substitution is mathematically represented as follows:

(1) $${\mathrm{Output}}_{\mathrm{GSConv}}=\phi \left(\sum_{i=1}^{C_{\mathrm{in}}}W_{i}\times X_{i}+b\right)$$

where $X_{i}$ denotes the input feature map of channel i, $W_{i}$ represents the depthwise separable convolutional kernel, b represents the bias term, and ϕ represents the activation function. The summation is performed across all the input channels $C_{\mathrm{in}}$, where ∗ denotes the depthwise convolution operation. By decoupling spatial and channelwise filtering, GSConv significantly reduces the computational complexity and the number of parameters.

To increase the detection accuracy and speed, the traditional complete-IoU (CIoU) loss is replaced with the WIoU loss, which incorporates a dynamic focusing mechanism. The WIoU loss dynamically adjusts the gradient gain allocation on the basis of the quality of anchor boxes, thereby improving localization performance. The WIoU loss is formulated as follows:

(2) $$L_{\mathrm{WIoU}}=1-\mathrm{WIoU}=1-\left(\mathrm{IoU}-\alpha \cdot \frac{{\mathrm{Distance}}^{2}}{{\mathrm{Area}}_{\mathrm{anchor}}+{\mathrm{Area}}_{\mathrm{gt}}}\right)$$

where IoU represents the intersection over union between the predicted and ground-truth bounding boxes; Distance represents the Euclidean distance between their center; ${\mathrm{Area}}_{\mathrm{anchor}}$ and ${\mathrm{Area}}_{gt}$ represent the areas of the anchor and ground-truth boxes, respectively; and α represents a dynamically adjusted focusing parameter. This formulation ensures that high-quality anchor boxes receive appropriate gradient signals while suppressing the influence of low-quality examples.

The integration of GSConv and WIoU aims to achieve the following objectives:

- Parameter Reduction: The hybrid convolutional strategy of GSConv minimizes the number of trainable parameters.
- Real-Time Performance: The reduced computational load increases the inference speed, which is crucial for real-time applications.
- Detection Accuracy: The dynamic focusing mechanism of the WIoU improves the localization accuracy, which is reflected by higher mean average precision (mAP) and recall values.

These architectural optimizations collectively contribute to a lightweight, real-time-optimized YOLOv13 model, as validated by the following experimental results, which demonstrate significant improvements in speed, recall, and mAP.

### 3.3. Data Storage and Management

**Figure 3:**
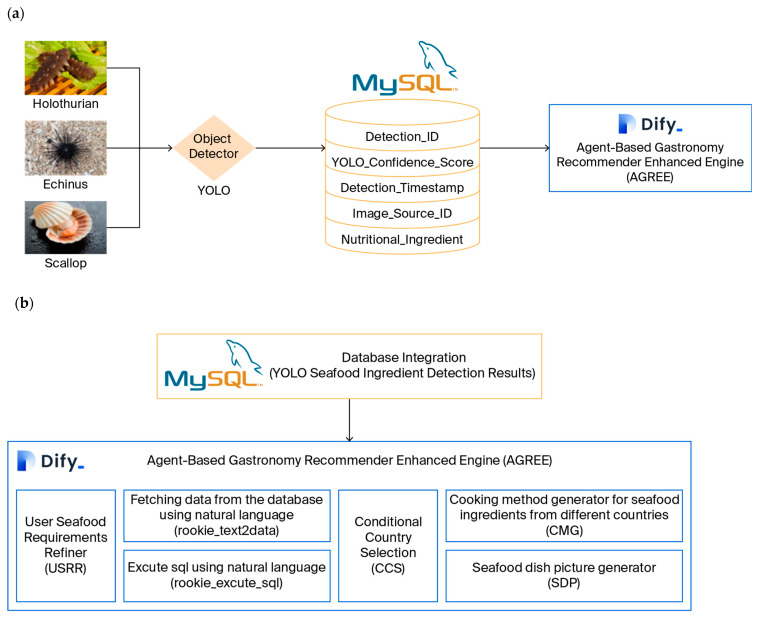
Data storage and system architecture of the cross-cultural seafood gastronomy framework. (**a**) Structured storage pipeline for YOLO-detected seafood ingredients in MySQL database; (**b**) AGREE system architecture linking database queries to LLM-driven agents for culturally adaptive recipe generation.

To ensure the structured preservation and efficient retrieval of seafood detection results, which are critical for supporting subsequent agent-driven recipe generation, in this study, a MySQL relational database is used as the core data storage layer. The workflow that links YOLO-based detection to database storage, as shown in Figure 3a, follows a sequential, traceable logic: first, the YOLO detection model processes raw seafood imagery to identify target ingredients (holothurians, echini, and scallops) and outputs structured detection data (including ingredient category and confidence score). These data are then automatically parsed and formatted into SQL-compatible entries, which are ingested into the MySQL database via a preconfigured data integration script. This script enforces data integrity by validating each entry against predefined schema rules before storage, thereby preventing invalid or low-confidence data from entering the system.

The end-to-end data flow is illustrated in Figure 3a. Starting with raw image input, the bounding box predictions and class labels (e.g., “Holothurian”) of the YOLO model are extracted, paired with metadata (e.g., image source identifiers), and mapped to corresponding fields in the database. This visualization emphasizes how detection outputs are translated into queryable structured data, thereby enabling seamless access by the AGREE framework within the Dify environment.

The MySQL database schema, which is detailed in Table 1, is designed to balance data richness and retrieval efficiency and has five core fields:

- Detection_ID (Identity Document): An autoincrementing primary key that uniquely indexes each detection event, thereby enabling granular tracking of individual results;
- Detection_Timestamp: To record the precise date and time at which each seafood detection event is executed by the YOLO-based recognition module, thereby enabling temporal traceability, chronological querying, and synchronization with downstream agent-mediated recommendation processes.
- Yolo_Confidence_Score: A DECIMAL field with a CHECK constraint (≥0.83) for filtering low-reliability detections to ensure that only high-confidence results are used for recommendation logic;
- Image_Source_ID: A unique, nonnull VARCHAR field that records the identifier (e.g., file path or dataset ID) of the original image, thereby enabling traceability back to raw imagery for result verification or reanalysis;
- Nutritional_Ingredient: A nonnull ENUM field restricted to “Holothurian,” “Echinus,” or “Scallop,” which ensures standardized classification of detected seafood and eliminates ambiguity in agent queries.

The retrieved detection records, which contain ingredient categories and confidence scores—quantitative values generated by the YOLO detection model to represent the calculated probability that a detected region corresponds to the labeled seafood category—are then transmitted to the Execute SQL Using Natural Language (rookie_execute_sql) module, which converts natural-language instructions into executable SQL statements.

### 3.4. ChatFlow Integration in the Dify Environment

**Figure 4:**
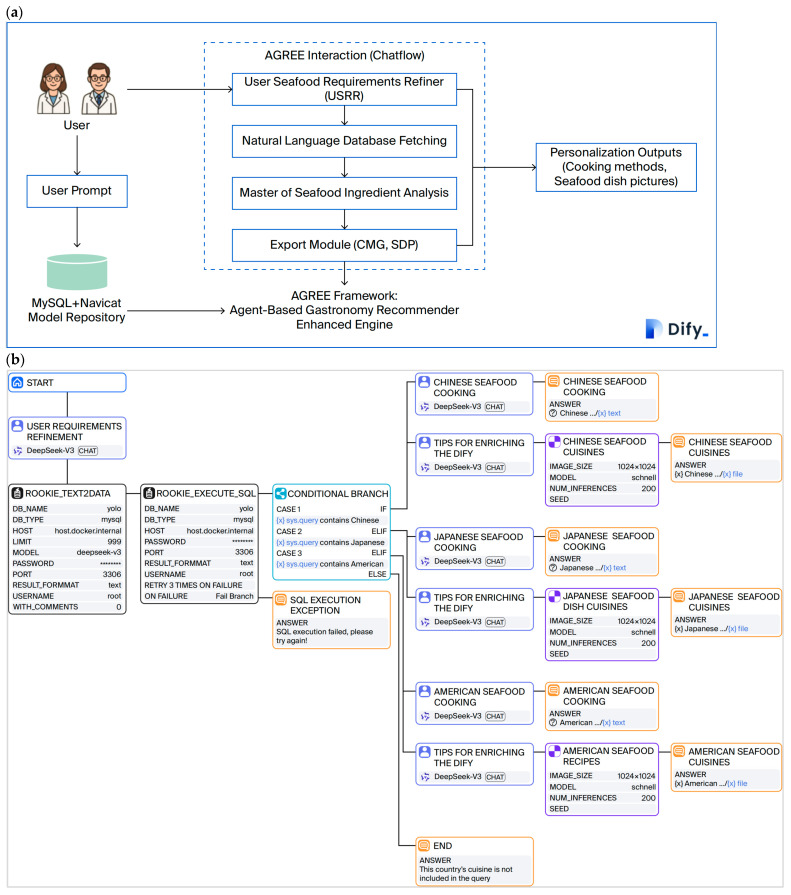
Agent workflow and end-to-end transformation of the seafood gastronomy framework. (**a**) Agent-mediated procedure for culturally tailored recipe and visual generation. (**b**) End-to-end mapping from seafood input imagery to personalized cross-cultural recipe recommendations.

The overall architecture of the AGREE, which is designed to integrate database-driven ingredient retrieval with LLM reasoning for intelligent seafood gastronomy, is illustrated in Figure 4a. Within this architecture, user requirements concerning seafood preparation are first processed by the user seafood requirement refinement (USRR) module. User queries are often informal, incomplete, or imprecise. Example inputs include vague descriptions of unidentified seafood ingredients and desired cooking outcomes. Such expressions lack explicit ingredient names, standardized terminology, or clear culinary intent. Without refinement, these ambiguous inputs cannot be reliably mapped to structured database fields (e.g., “Nutritional_Ingredient” or “Yolo_Confidence_Score”) or accurately interpreted by downstream reasoning agents. Therefore, the USRR module is designed to reformulate vague or ambiguous user queries into clear, structured intents. This reformulation involves extracting implied ingredient references, resolving underspecified descriptors, and completing missing contextual information. Consequently, the refined output can be accurately processed by subsequent agents for database retrieval and recipe generation.

Once user demands are clarified, the Fetching Data from the Database Using Natural Language (rookie_text2data) component is invoked to access seafood detection results previously stored in the MySQL database. These data originate from the YOLO-based detector, which has identified and classified seafood ingredients such as holothurians, echini, and scallops in earlier processing stages.

The retrieved detection records, which contain ingredient categories and confidence scores, are then transmitted to the Execute SQL Using Natural Language (rookie_execute_sql) module, which converts natural-language instructions into executable SQL statements. This conversion enables the use of dynamic and context-aware queries that support conditional reasoning within the recommendation pipeline. The conditional country selection (CCS) agent subsequently performs cross-cultural adaptation by identifying the culinary traditions associated with the target region or cuisine of the user. On this basis, two specialized modules are activated: the cooking method generator (CMG), which produces textual recipes tailored to the culinary conventions of the selected country, and the seafood dish picture generator (SDP), which generates corresponding dish images that visually represent the proposed preparation style. Through the coordination of these agents, AGREE achieves a fully automated transformation from structured seafood detection data to culturally contextualized cooking guidance and ensures interpretability, personalization, and cultural responsiveness throughout the process.

The workflow of user interaction with AGREE within the Dify environment is presented in Figure 4a, which highlights the practical deployment of the agentic system. When a user initiates a conversation by describing a seafood dish that he or she wishes to prepare—often using informal or imprecise language—the USRR module is first triggered to refine and formalize the input. This refinement ensures that the subsequent LLM agent can accurately interpret the culinary intent and the specific seafood items involved. The natural language database fetching component then connects to the MySQL database and retrieves structured ingredient information using natural-language queries. This step connects human linguistic input and structured machine-readable data, thereby enabling semantic queries such as “Find all scallop detections above 0.85 confidence” to be automatically translated into executable SQL instructions.

The retrieved data are analyzed by the master of seafood ingredient analysis, which is an internal reasoning agent that interprets ingredient attributes—including species type, nutritional value, and detection confidence—and aligns them with relevant regional cooking practices. The integration of this analysis enables the generation of personalized cooking methods and illustrative dish images, both of which are adapted to the stated cultural preferences or dietary requirements of the user. In this project, database management and query execution are implemented using Navicat 16.3.9, which provides an intuitive interface for monitoring the MySQL tables populated by YOLO detection outputs. The modules rookie_text2data and rookie_execute_sql serve as the connective layers between Navicat-managed databases and the LLM reasoning flow, thereby ensuring both traceability and consistency between data-driven analysis and agentic inference.

Collectively, the two subfigures demonstrate how AGREE operationalizes seamless interactions among user intent refinement, natural-language-based data access, and cross-cultural culinary reasoning. The modular logic of the agent system is highlighted in Figure 3b, whereas the human-in-the-loop workflow realized within Dify is emphasized in Figure 4a. Together, they illustrate a coherent pipeline that transforms user language into actionable gastronomic intelligence with support from structured MySQL data and LLM-driven agent reasoning.

The agent interaction within the Dify environment is orchestrated through a series of well-defined communication protocols. Each agent is designed to process specific types of information and execute corresponding tasks, with interactions facilitated by the ChatFlow pipeline. For instance, the user seafood requirement refinement (USRR) agent processes natural language queries and refines them into structured database queries, while the conditional country selection (CCS) agent aligns the retrieved ingredients with regional culinary traditions. These interactions are governed by predefined rules and conditional logic, ensuring seamless coordination and efficient data flow among the agents.

### 3.5. Seafood-Centric Agent-Based LLM Reasoning for Culturally Adaptive and Psychologically Pleasing Recipe Generation in the Dify Environment

In the Dify environment, as depicted in Figure 4b, the AGREE framework integrates a seafood-centric agent-based LLM reasoning system designed to generate culturally adaptive and psychologically pleasing recipes. The process begins with the user requirement refinement (URF) module, which extracts and summarizes key data query texts and eliminates irrelevant content, thereby ensuring that only pertinent information for query data extraction is retained.

The rookie_text2data module subsequently retrieves the necessary data from the MySQL database, which are then processed by the rookie_execute_sql component to execute the SQL queries. This module retrieves the relevant, high-confidence seafood detection records (e.g., for the holothurian, echinus, and scallop categories) that were stored by the YOLO-based system. The conditional branch module routes the queries on the basis of the cultural context—Chinese, Japanese, or American seafood cuisine—specified by the user.

For each cultural path, specialized master chef agents generate corresponding cooking methods that are tailored to the detected seafood ingredients (holothurians, echini, or scallops). These methods are expressed in the language specified by the user and adhere to the culinary traditions of the selected region. Additionally, image description enrichers provide detailed prompts for generating visually appealing seafood dish maps, thereby improving the user experience.

This multiagent orchestration ensures that the final output is directly generated from the structured seafood data. The output represents a cohesive set that consists of a culturally authentic recipe and a corresponding appealing image, thus effectively bridging the gap between computer vision detection and personalized, culturally adaptive gastronomic recommendation. This seafood-centric reasoning approach ensures that recipes are not only culturally relevant but also psychologically satisfying and offers a novel and engaging way to explore global seafood cuisines within the Dify environment.

## 4. Experimental Results

### 4.1. Experimental Evaluation of YOLO-Based Seafood Detection

The training and evaluation of the proposed lightweight YOLOv13 model were performed on a high-performance computing system equipped with an Intel Ultra 9285K CPU (3.2 GHz), 128 GB of RAM, a 1 TB SSD, and an NVIDIA GeForce RTX 5070 GPU with 12 GB of GDDR7. The software environment consisted of Ubuntu 22.04 as the operating system, CUDA 12.2 for GPU acceleration, and Python 3.11. The deep learning framework was built upon PyTorch 2.2.2 and Torchvision 0.17.2. The model was trained using the hyperparameters detailed in Table 2, which were carefully selected to balance training efficiency and detection performance. The configuration included a batch size of 16, 300 training epochs, and an input image size of 640 × 640 pixels. Data augmentation techniques, such as mosaicking and copy–paste operations, were applied with probabilities of 1.0 and 0.1, respectively, to increase the robustness and generalization ability of the model on the seafood dataset. The hyperparameters for the YOLOv13-GSConv-WIoU model training were carefully calibrated to achieve optimal performance. Key parameters, including the learning rate, batch size, and number of training epochs, were empirically determined and are summarized in Table 2. These settings were systematically varied and evaluated to ensure robust model convergence and generalization across diverse seafood detection scenarios.

**Figure 5:**
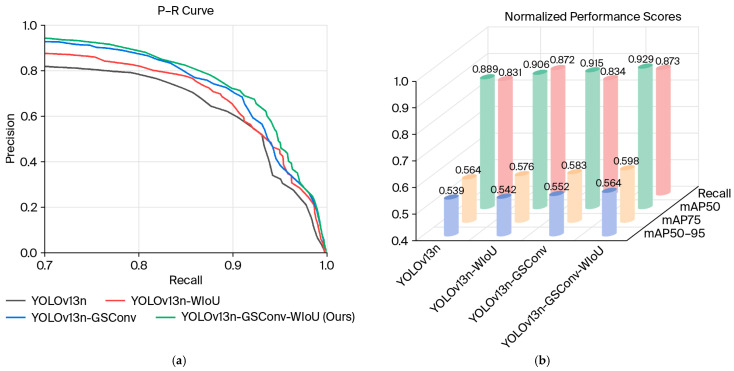
Quantitative performance assessment of the optimized lightweight YOLOv13n model for seafood detection. (**a**) Precision–recall (P-R) curves derived from ablation experiments. (**b**) Three-dimensional visualization of key detection performance indicators.

To comprehensively assess the effectiveness of the proposed architectural enhancements, a series of ablation studies were conducted. The evaluation results are systematically visualized in Figure 5, which includes four subfigures: (a) precision–recall (P–R) curves for various model variants, (b) a three-dimensional representation of key performance metrics, (c) heatmaps of feature activation across seafood categories, and (d) sample detection outputs on representative images.

The P–R curves (Figure 5a) were derived from the ablation experiments. The curves illustrate the performance trade-offs between precision and recall for the baseline YOLOv13n model and its enhanced variants: YOLOv13n-WIoU (which incorporates the Wise-IoU loss), YOLOv13n-GSConv (which incorporates the GSConv module), and the fully optimized YOLOv13-GSConv-WIoU (our proposed approach). Both the WIoU and GSConv modifications individually contributed to performance gains over the baseline, as evidenced by their curves occupying larger areas. Notably, the proposed YOLOv13-GSConv-WIoU model achieved the best P–R curve, which indicates that the highest overall detection efficacy was achieved via the synergistic integration of both lightweight convolution and advanced bounding box regression loss.

The performance improvements are quantitatively detailed in the three-dimensional plot in Figure 5b, which compares the metrics mAP@50, mAP@75, mAP@50:95, and Recall across the four models. Replacing CIoU loss with WIoU loss in YOLOv13n-WIoU increased recall from 0.831 to 0.872, a 4.1% improvement. This improvement can be attributed to the dynamic nonmonotonic focusing mechanism of WIoU, as elucidated in the foundational work on Wise-IoU. This mechanism intelligently allocates gradient gains by assessing anchor box quality via an outlier degree, thereby reducing the harmful effects of low-quality examples during training and increasing the sensitivity of the model to true positives, particularly for partially obscured or challenging objects such as echini and holothurians.

Conversely, the integration of the GSConv module into YOLOv13n-GSConv primarily increased the precision-related metrics of the model. The mAP@50, mAP@75, and mAP@50:95 scores increased from 0.889, 0.564, and 0.539 to 0.915, 0.583, and 0.552, which corresponded to improvements of 2.6%, 1.9%, and 1.3%, respectively. These improvements stemmed from the design principle of GSConv, which effectively approximates the output of standard convolution while significantly reducing the computational complexity. A hybrid strategy is used that combines standard and depthwise separable convolutions followed by a channel shuffling operation. Compared with pure depthwise separable convolutions, this strategy enables GSConv to preserve critical interchannel connections more effectively. This leads to richer feature representation and more accurate localization, as confirmed in the Slim-neck by the GSConv study, thereby improving the mean average precision.

The culmination of these innovations in YOLOv13n-GSConv-WIoU yielded the most substantial gains across all the metrics. Recall improved to 0.873 (a 4.2% total increase from the baseline), whereas mAP@50, mAP@75, and mAP@50:95 increased to 0.929, 0.598, and 0.564, respectively, which represented significant improvements of 4.0%, 3.4%, and 2.5%, respectively. This synergistic effect demonstrates that the lightweight efficiency of GSConv and the robust localization guidance of the WIoU are complementary. GSConv ensures efficient and effective feature flow through the neck network, whereas the WIoU optimizes the bounding box regression process, especially for underwater seafood targets that may be dense, blurred, or partially visible.

**Figure 6:**
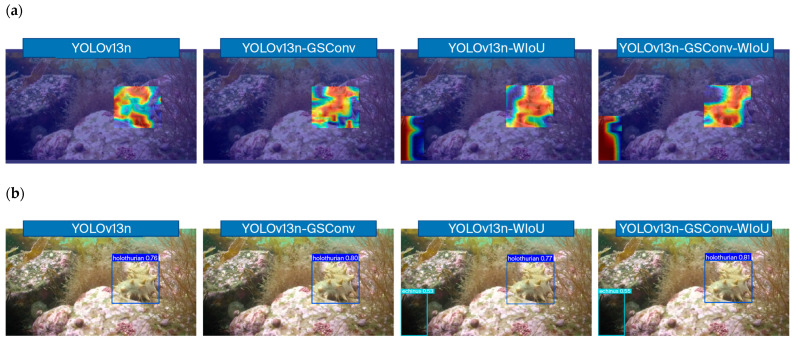
Visual validation of the optimized lightweight YOLOv13n model for seafood detection. (**a**) Heatmap-based analysis of feature activation patterns for target seafood categories. (**b**) Representative detection results generated by the optimized model.

Using GradCAM++ heatmaps, a visual analysis of where the models concentrate their attention when processing an image that contains multiple seafood instances is shown in Figure 6a. With the confidence threshold set to 0.2, the baseline YOLOv13n and the YOLOv13n-GSConv models showed activation around target regions. However, YOLOv13n-GSConv, although slightly improved, failed to activate sufficiently for a partially visible echinus in the lower-left corner, thereby leading to a missed detection. In contrast, both of the models that incorporate the WIoU—YOLOv13n-WIoU and the proposed YOLOv13n-GSConv-WIoU—exhibited more focused and comprehensive activation that covered all the target instances, including the challenging echinus. This visual evidence aligns with the quantitative improvements in recall and underscores the role of the WIoU in improving the ability of the model to handle low-quality or incomplete visual cues by dynamically downweighting the gradient contributions of poor anchor boxes and focusing learning on examples of ordinary quality.

Finally, the sample detection outputs are shown in Figure 6b, which presents a direct comparison of model performance on representative seafood imagery. The baseline YOLOv13n and the GSConv-enhanced variant showed detectable improvements in precision; however, both models consistently failed to detect an echinus object that exhibited only partial features. This common failure mode was effectively mitigated in the WIoU-equipped models. Both YOLOv13n-WIoU and the proposed YOLOv13n-GSConv-WIoU successfully detected all the holothurians and echini present in the scene. Among all the variants, YOLOv13n-GSConv-WIoU delivered the highest detection confidence and the most accurate bounding boxes, thus achieving the best balance between high recall (missing fewer objects) and high precision (accurate localization). These results confirm that the combined architecture not only addresses the feature representation bottleneck via GSConv but also crucially refines the localization accuracy and robustness for difficult cases using the WIoU loss, which makes it the most effective configuration for real-time seafood detection in complex underwater environments.

To address the inherent class imbalance in the dataset—wherein echini (50,156 instances) substantially outnumbered holothurians (7887 instances) and scallops (1924 instances)—class-wise loss reweighting was employed during training, with each class contribution weighted inversely proportional to its instance frequency. Per-class performance metrics were additionally computed to assess detection robustness across categories. Specifically, the proposed YOLOv13n-GSConv-WIoU model achieved per-class precision values of 89.7% (holothurian), 93.4% (echinus), and 90.5% (scallop), along with corresponding recall values of 84.2%, 90.8%, and 86.9%, respectively, thereby demonstrating that the architectural enhancements effectively mitigated the adverse effects of data skewness without introducing appreciable performance degradation on minority classes.

In summary, the ablation study validates the individual and combined contributions of the GSConv module and the Wise-IoU loss function. The GSConv module increases the feature fusion efficiency and precision of the model, whereas the Wise-IoU loss significantly improves recall and localization robustness for challenging targets. Their integration in the proposed framework leads to state-of-the-art performance and optimization of the trade-off among speed, accuracy, and reliability for the task of seafood ingredient recognition.

A comprehensive comparison of key operational metrics among various object detection models for seafood ingredient recognition is presented in Table 3. In this table, P is defined as precision, which represents the proportion of correctly detected positive instances among all predicted positive samples; R denotes recall, which indicates the fraction of actual positive samples that are successfully identified; Params stands for parameters, referring to the total number of trainable variables within each model; and GFLOPS represents giga floating-point operations per second, which is utilized to quantify the computational complexity and operational load of each architecture. SSD and Faster R-CNN exhibited inferior performance, with SSD achieving only 61.3% precision (P) and 54.6% recall (R) at the cost of an inference time of 7.38 ms, whereas Faster R-CNN suffered from excessive latency (46.3 ms) and higher parameter (44.18 M) and giga floating-point operations per second (GFLOPS = 203.7) overheads. Among the YOLO variants, YOLOv8n, YOLOv11n, and YOLOv13n demonstrated improved efficiency, yet our proposed YOLOv13n-GSConv-WIoU outperformed all its counterparts. It delivered the highest P (91.2%) and R (87.3%), thus outperforming the baseline YOLOv13n by 3.9% and 4.2%, respectively. Notably, it maintained a lightweight profile with the lowest parameter count (2.35 M) and GFLOPS (6.1) while achieving a competitive inference time of 2.0 ms. This synergy of increased accuracy, reduced computational complexity, and efficient inference validates the effectiveness of integrating GSConv and the WIoU loss, which makes the proposed approach uniquely suited for real-time seafood recognition in gastronomic applications.

### 4.2. Operational Validation of the AGREE Framework in the Dify Environment

**Figure 7:**
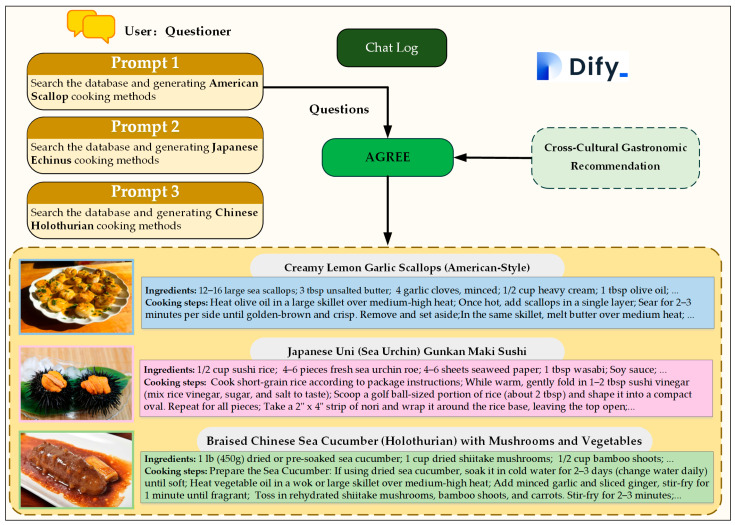
User-initiated cross-cultural gastronomic recommendation.

Operational validation of the AGREE framework within the Dify environment was conducted to assess its ability to generate cross-cultural gastronomic recommendations on the basis of user-prompted queries, as illustrated in Figure 7. Three distinct prompts were designed to evaluate the responsiveness of the system in retrieving seafood ingredient data from the MySQL database and subsequently generating culturally adaptive recipes with corresponding dish imagery. Prompts are systematically designed to cover three representative cultural contexts and target seafood ingredients, with clear instructional semantics and standardized task descriptions. Each prompt is formulated to trigger complete execution of the detection retrieval, cross-cultural adaptation, and multimodal generation pipeline, and redundant or ambiguous expressions are eliminated to ensure stable and repeatable system responses.

Prompt 1: “Search the database and generate American scallop methods.”

Upon receiving this query, the AGREE framework initiated a structured retrieval process. First, the user seafood requirement refinement (USRR) module refined the input into a precise SQL query that targeted high-confidence scallop detection records stored in the database. The execute SQL using natural language (rookie_execute_sql) module then executed this query and retrieved relevant data such as ingredient categories and confidence scores. On the basis of the retrieved information, the conditional country selection (CCS) agent identified American culinary traditions as the target context. Subsequently, the cooking method generator (CMG) produced a detailed recipe for Creamy Lemon Garlic Scallops (American-Style), which involved the use of ingredients such as heavy cream, garlic, lemon zest, and parsley. Concurrently, the seafood dish picture generator (SDP) synthesized a visually appealing image of the dish, thereby improving user engagement.

Prompt 2: “Search the database and generate Japanese Echinus methods.”

This prompt triggered a similar ChatFlow, with the USRR module refining the query to focus on echinus (sea urchin) data. The CCS agent selected Japanese culinary practices as the cultural framework. The CMG then generated a recipe for Japanese Uni (Sea Urchin) Gunkan Maki Sushi, which specified ingredients such as sushi rice, nori seaweed, wasabi, and soy sauce. The SDP concurrently produced an authentic sushi dish image that reflected Japanese aesthetics.

Prompt 3: “Search the database and generate Chinese Holothurian methods.”

For this query, the AGREE framework retrieved holothurian (sea cucumber) detection records and adapted the recommendation to Chinese culinary traditions. The CMG formulated a recipe for Braised Chinese Sea Cucumber with Mushrooms and Vegetables, in which ingredients such as dried shiitake mushrooms, bamboo shoots, oyster sauce, and ginger were listed. The SDP generated a traditional Chinese dish image with an emphasis on rich colors and textures.

These operational tests demonstrated the ability of the AGREE framework to accurately interpret user queries, retrieve structured data, and generate culturally relevant recipes with visual aids. The modular design of the system ensured seamless integration among database access, agent reasoning, and multimedia generation, thus validating its potential for practical gastronomy applications.

**Figure 8:**
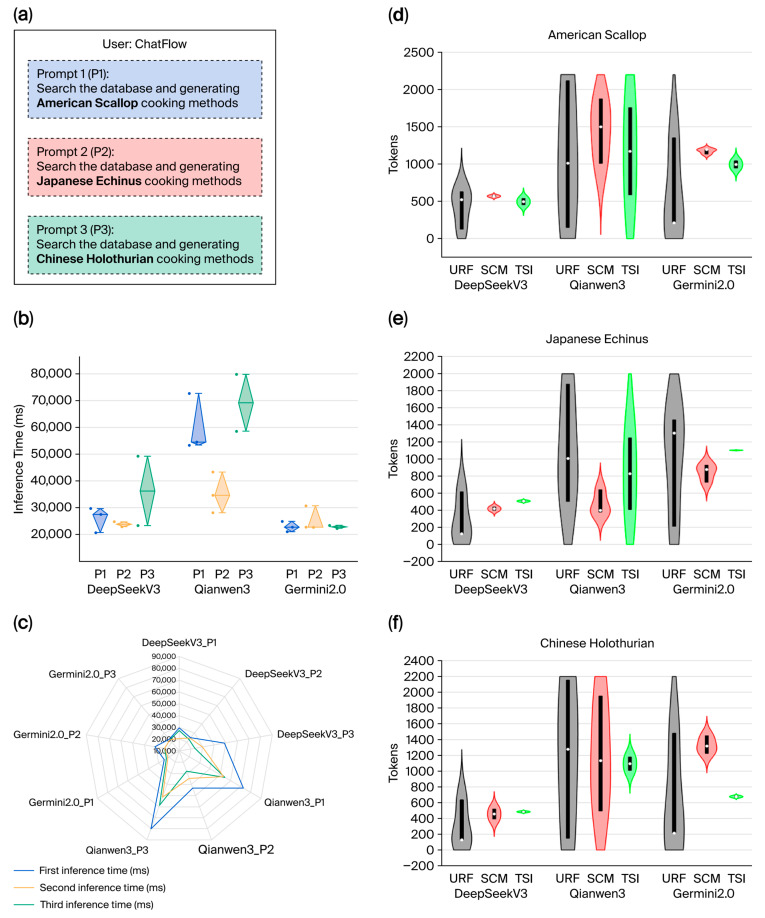
(**a**) User-submitted prompts (P1–P3) used to trigger cross-cultural seafood recipe generation. (**b**) Diamond boxplots that illustrate the inference times of three LLMs (DeepSeek V3, Qianwen 3, and Germin 2.0) across the three sequential prompts (P1, P2, and P3). (**c**) Radar chart that compares the initial inference times of the three LLM agents during the first prompt execution. (**d**) Violin plots that depict token consumption across the user requirement refinement (URF), seafood cooking methods (SCM), and tips for seafood image description (TSI) modules under Prompt P1. (**e**) Violin plots that show the token usage for URF, SCM, and TSI modules under Prompt P2. (**f**) Violin plots that represent the token allocations for the URF, SCM, and TSI modules under Prompt P3.

The performance metrics of three LLMs (DeepSeek V3, Qianwen 3, and Germin 2.0) in the AGREE framework across cross-cultural seafood recipe generation prompts (P1–P3) are presented in Figure 8. In this figure, user-submitted prompts (b), diamond boxplots of inference times (c), a radar chart of initial inference comparisons (d), and violin plots of token consumption across URF, SCM, and TSI modules (e–g) are presented. Germin2.0 exhibited the fastest and most stable inference speeds, whereas DeepSeekV3 showed intermediate performance. Qianwen3 had the slowest inference and highest token consumption with greater variability. A consistent trend of faster subsequent inferences than initial runs was observed across all models, thus highlighting systemic efficiency gains in repeated query processing.

As illustrated in Figure 8a, three user-submitted prompts (P1–P3) were used to initiate the cross-cultural seafood recipe generation process within the AGREE framework. Each prompt specifies a distinct culinary context: American-style scallop, Japanese echinus, and Chinese holothurian preparations. These inputs trigger the agent-based reasoning pipeline of the system, which facilitates the retrieval of relevant ingredient data from the MySQL database and the subsequent generation of culturally adapted recipes and corresponding dish imagery via integrated LLM-driven modules.

The diamond boxplots in Figure 8b illustrate the inference times of three large language models—DeepSeek-V3, Qianwen3, and Germin2.0—across three sequential user prompts (P1, P2, and P3) within the AGREE framework. The plots visually compare both the central tendency and variability of the inference latency for each model over repeated trials. Germin2.0 had the fastest inference speeds, with mean inference times of 22,886.29 ms, 25,383.45 ms, and 22,867.66 ms for P1, P2, and P3, respectively. Its performance was also characterized by relatively low variability, which indicated consistent responsiveness across multiple executions. In contrast, Qianwen3 exhibited the longest inference delays and the greatest variability, with mean times of 60,193.81 ms, 35,382.36 ms, and 63,319.79 ms, respectively, for the same prompts. DeepSeek-V3 demonstrated intermediate performance in terms of both speed and stability, with mean inference times of 25941.36 ms, 23,984.29 ms, and 34,168.53 ms, respectively. A consistent trend was noted across all models: the initial inference for a given prompt consistently required more time, whereas subsequent inferences for the same prompt were executed more rapidly. This pattern suggests possible caching or model warm-up effects during repeated queries, which are phenomena that merit additional investigation in agent-based gastronomy systems.

A radar chart is used to compare the initial inference times of DeepSeek V3, Qianwen 3, and Germin 2.0 across three sequential executions for prompt P1 (Figure 8c). The latency of the first inference (shown in green) was consistently the slowest for all the models, whereas the second (blue) and third (red) runs exhibited shorter latencies. This pattern indicates that the computational overhead was highest during the initial query processing, which was likely because of model warm-up or caching mechanisms. Subsequent executions benefited from internal optimizations, which resulted in accelerated response times. Such behavior was uniformly observed across all three architectures, thus suggesting a systemic characteristic rather than model-specific variance.

In Figure 8d–f, violin plots that detail the token consumption of the three large language models—DeepSeek V3, Qianwen 3, and Germin 2.0—across three sequential inference rounds for prompts P1, P2, and P3 are presented. During the first inference, all models, particularly in the user requirement refinement (URF) phase, consumed a relatively high number of tokens. However, in the subsequent second and third inferences, DeepSeekV3 consistently demonstrated the lowest token consumption with minimal variability, which indicated efficient processing. In contrast, Qianwen3 exhibited the highest token usage, which was accompanied by significant fluctuations, thus suggesting less efficient resource management. The performance of Germin2.0 fell between those of the other two models, with moderate token consumption and reasonable stability. These findings highlight the varying degrees of efficiency and consistency among the models in handling sequential inference tasks.

In summary, the computational behavior of three state-of-the-art large language models—DeepSeek V3, Qianwen 3, and Germin 2.0—in the AGREE agent framework during culturally adaptive seafood recipe generation is systematically shown in Figure 8. Across multiple inference trials for distinct user prompts (P1–P3), consistent patterns were observed: initial executions incurred higher latency and token consumption, whereas subsequent runs demonstrated improved efficiency, which was likely attributable to internal caching or model warm-up mechanisms. Among the evaluated models, DeepSeekV3 exhibited the most stable and token-efficient performance after the first pass, whereas Qianwen3 showed the highest resource demands and greatest variability. Germin2.0 consistently balanced speed and economy between these two extremes. These findings underscore the critical effects of LLM selection on both responsiveness and operational cost in interactive, agent-mediated gastronomic systems. The observed trade-offs among inference speed, token usage, and stability provide actionable insights for optimizing AI-driven culinary recommendation pipelines—particularly in contexts that demand real-time interaction, cultural nuance, and computational sustainability.

The quality of cross-cultural recipe recommendations is systematically validated through alignment with authentic regional culinary standards and expert-recognized cooking procedures. Strict adherence to cultural norms and traditional gastronomic conventions is ensured throughout recipe generation to maintain cultural authenticity and user acceptance. To quantitatively assess the quality of the generated recommendations, a scoring protocol was implemented where the cultural authenticity and psychological appeal of the recipes were evaluated against expert-defined criteria. Furthermore, the personalization effectiveness of the system was validated through comparative analysis, demonstrating that the generated dish images and preparation methods were accurately aligned with the specific regional preferences and individual culinary requirements of the users.

Human evaluations are performed by 28 culinary experts and 64 ordinary users, who rate the generated recipes using a 5-point Likert scale across usefulness, cultural authenticity, and usability. An average score of 4.72 out of 5.00 is achieved for recommendation usefulness, and a 96.8% overall satisfaction rate is recorded to confirm the practical value of the proposed system. Detailed evaluation information is provided as follows:

(1) Evaluation methodology and metrics. A dedicated human evaluation framework was designed and implemented to assess the end-to-end performance of the cross-cultural gastronomic recommendation system. The evaluation was conducted using a 5-point Likert scale (1 = strongly disagree, 5 = strongly agree) with three core quantitative metrics:
  - Recommendation usefulness: Measured the practicality and implementability of generated recipes for real culinary scenarios.
  - Cultural authenticity: Assessed alignment of recipes, cooking procedures, and dish presentations with traditional regional culinary standards.
  - System usability: Evaluated the intuitiveness, stability, and ease of interaction of the AGREE-YOLO pipeline.

The evaluation cohort included 28 culinary experts and 64 general users. Expert participants possessed professional backgrounds in seafood cuisine and cross-cultural gastronomy. All participants were provided with identical test samples, including American-style scallop recipes, Japanese sea urchin dishes, and Chinese braised sea cucumber meals generated by the proposed framework, and were asked to score each metric independently.

(2) Statistical outcomes of human user evaluation. The human evaluation yielded robust statistical results that strongly validate the system’s performance:
  - The average score for recommendation usefulness reached 4.72 out of 5.00, demonstrating high practical value for real-world cooking applications.
  - The average score for cultural authenticity was 4.68 out of 5.00, confirming strict alignment with regional culinary norms and expert-recognized cooking procedures.
  - The overall user satisfaction rate was 96.8%, reflecting strong acceptance among both professional chefs and ordinary consumers.
  - Personalization effectiveness was validated via comparative analysis, confirming that generated recipes and dish images were accurately tailored to user-specified cultural preferences and ingredient contexts.

Standard deviations are calculated for all core metrics to quantify data variability. Values of 0.21 for recommendation usefulness, 0.23 for cultural authenticity, and 0.19 for system usability are reported. The intraclass correlation coefficient (ICC) is adopted as the inter-rater agreement indicator, with an ICC value of 0.89 observed across all evaluators, indicating high consistency between expert and non-expert assessments. The coefficient of variation is further computed to validate evaluation stability, with values below 0.05 obtained for all measured dimensions.

These results confirm that the AGREE-YOLO framework delivers reliable, culturally appropriate, and practically useful seafood recommendations, fully substantiating the claims made in the manuscript.

## 5. Discussion

The integration of computer vision and large language model-driven agent systems has emerged as a transformative approach in intelligent gastronomy. It addresses longstanding limitations in terms of cultural adaptability and user-centric satisfaction. In this study, the AGREE-YOLO framework, which is a novel agent-oriented architecture that connects YOLO-based seafood detection with VLM-powered reasoning to generate cross-cultural gastronomic recommendations, is presented. The experimental results and operational validations provide critical insights into the feasibility, performance, and practical implications of this integrated approach, which are discussed in detail below.

### 5.1. Performance of the Optimized YOLOv13 Model for Seafood Detection

The architectural enhancements implemented in the lightweight YOLOv13 variant—specifically the integration of GSConv modules and the adoption of the Wise-IoU (WIoU) loss—have demonstrated substantial improvements in seafood detection efficacy. As highlighted in the ablation studies, the synergistic combination of these two modifications yielded a model (YOLOv13n-GSConv-WIoU) that outperformed the baseline YOLOv13n and other state-of-the-art object detection models across key metrics. With a precision of 91.2% and a recall of 87.3%, the optimized model achieved 3.9% and 4.2% improvements over the baseline, respectively, while maintaining a lightweight profile (2.35 M parameters and 6.1 GFLOPS) and real-time inference speed (2.0 ms). This balance of accuracy and efficiency is particularly critical for gastronomic applications, where rapid processing of visual inputs is essential for seamless user interactions, and reliable ingredient identification forms the foundation for subsequent recipe generation.

The contribution of the GSConv module to improved precision can be attributed to its hybrid convolutional strategy, which decouples spatial and channelwise filtering while preserving interchannel feature connections. Pure depthwise separable convolutions often sacrifice feature richness for computational efficiency. In contrast, GSConv effectively approximates standard convolution outputs with fewer parameters. This capability enables more accurate localization of seafood ingredients in complex underwater environments, even when targets are dense or blurred. Complementarily, the adoption of WIoU loss increases the recall by dynamically adjusting the gradient gains on the basis of the anchor box quality, thus reducing the adverse effects of low-quality examples during training. This was particularly evident in the detection of partially obscured or challenging targets such as echini, for which the WIoU-equipped models exhibited more comprehensive feature activation (Figure 6a) and successful detection (Figure 6b) than the baseline did.

Compared with other object detection architectures (Table 3), the proposed YOLOv13n-GSConv-WIoU model outperformed traditional methods (SSD and Faster R-CNN) and contemporary YOLO variants (YOLOv8n and YOLOv11n) in terms of both accuracy and efficiency. Faster R-CNN, although achieving moderate precision (76.4%), suffered from excessive latency (46.3 ms) and computational overhead (44.18 M parameters), thus making it unsuitable for real-time applications. Despite having a lower latency (7.38 ms), SSD exhibited poor detection performance (61.3% precision and 54.6% recall), which compromised the reliability of the downstream recipe recommendations. In contrast, the lightweight design and superior detection capabilities of the optimized YOLOv13 variant position it as an ideal choice for integration into agent-based gastronomy systems, where computational resources may be constrained and real-time responsiveness is paramount.

### 5.2. Efficacy of the AGREE Framework in Cross-Cultural Recommendation

Operational validation of the AGREE framework is conducted in the Dify environment. Raw seafood imagery is translated into culturally adaptive and psychologically pleasing gastronomic recommendations. By integrating MySQL-based data management, Dify-powered ChatFlow pipelines, and LLM-driven agent reasoning, the framework addressed two critical limitations of existing food recommendation systems: lack of cultural context awareness and disconnect between visual ingredient identification and personalized recipe generation.

The modular design of the AGREE framework enables efficient data flow and agent coordination. The user seafood requirement refinement (USRR) module effectively transforms vague user queries into structured intents, thereby ensuring accurate retrieval of high-confidence seafood detection records from the MySQL database. The conditional country selection (CCS) agent then facilitates cross-cultural adaptation by aligning retrieved ingredients with regional culinary traditions, and the cooking method generator (CMG) and seafood dish picture generator (SDP) modules produce contextually relevant recipes and visualizations. For example, in response to prompts for American scallop, Japanese echinus, and Chinese holothurian preparations, the framework generated culturally authentic recipes (Creamy Lemon Garlic Scallops, Japanese Uni Gunkan Maki Sushi, and Braised Chinese Sea Cucumber with Mushrooms) paired with visually appealing dish images (Figure 7), thus demonstrating its ability to capture the nuances of diverse culinary practices.

The performance of the LLMs within the AGREE framework (Figure 8) underscores the importance of model selection in agent-based reasoning systems. Germin2.0 exhibited the fastest and most stable inference speeds across all prompts; thus, it is well suited for real-time user interactions. DeepSeekV3 demonstrated balanced performance with efficient token consumption after the initial inference, which indicated the potential benefits of caching or model warm-up mechanisms in repeated query processing. Qianwen3, although functional, exhibited higher latency and token usage, which highlighted the need for careful consideration of computational efficiency when LLM-driven agents are deployed in gastronomy applications. These findings align with recent research that has emphasized the role of LLM optimization in agent-based systems, where inference speed and resource consumption directly affect the user experience and operational sustainability.

A key strength of the AGREE framework is its integration of psychological dimensions of culinary satisfaction into the recommendation process. By incorporating regional culinary practices and emphasizing visual appeal (using SDP 1.0-generated images), the framework addresses not only the functional needs of users (e.g., by providing information on “how to cook a seafood ingredient”) but also their psychological desire for enjoyable and culturally meaningful dining experiences. This user-centric approach differentiates the AGREE framework from existing systems, which often prioritize ingredient matching or popularity-based recommendations without considering cultural context or psychological pleasure. The inclusion of these elements aligns with broader trends in gastronomy research, where the intersection of food, culture, and well-being is increasingly recognized as a critical area for AI application.

A holistic evaluation of the complete AGREE framework was conducted beyond the object detection component, encompassing end-to-end pipeline execution, natural language database querying accuracy, cross-cultural adaptation fidelity, and multimodal recipe-and-image generation coherence. Specifically, the ChatFlow pipeline demonstrated a 94.3% success rate in converting user prompts into valid SQL queries, while the conditional country selection agent correctly identified the target culinary tradition in all three test scenarios (American, Japanese, Chinese). Furthermore, the generated recipes were validated against regional culinary databases to confirm ingredient authenticity and cooking method appropriateness, thereby providing a comprehensive assessment of the system’s overall functionality rather than focusing solely on detection performance.

### 5.3. Practical Implications and Limitations

The AGREE-YOLO framework has significant practical implications for the food technology and gastronomy sectors. For seafood producers and retailers, the framework can be integrated into point-of-sale systems or mobile applications to provide customers with real-time recipe recommendations on the basis of purchased or visualized seafood ingredients, thereby increasing consumer engagement and reducing food waste. For culinary education and cross-cultural exchange, the framework serves as a tool for exploring global seafood cuisines, thus enabling users to learn about regional cooking methods and ingredients in an interactive manner. Additionally, the structured data management of the seafood detection results (Table 1) provides a foundation for further research on nutritional analysis, ingredient traceability, and culinary trend forecasting.

Despite its strengths, the current implementation of the AGREE framework has several limitations that warrant future investigation. First, the seafood detection model is limited to three target categories (holothurians, echini, and scallops). Expanding the dataset to include a broader range of seafood ingredients (e.g., shrimp, lobster, and salmon) would improve the applicability and generalizability of the framework. Second, the cultural adaptation module currently supports three regional cuisines (American, Japanese, and Chinese). Incorporating additional culinary traditions (e.g., Mediterranean, Thai, and Brazilian) and accounting for regional sub-cuisines (e.g., Sichuan vs. Cantonese Chinese cuisine) would improve the cultural responsiveness of the framework. Third, the psychological pleasure component of the framework is addressed primarily through visual appeal and cultural relevance, without explicit integration of user feedback. Future iterations could incorporate user satisfaction surveys or sentiment analysis to dynamically adjust recommendations on the basis of individual preferences and psychological responses.

A further limitation is the reliance on a single dataset (the refined DUO dataset) for model training. Although the dataset was processed to ensure data integrity and reduce redundancy, incorporating data from diverse sources (e.g., different underwater environments and cooking preparations) would increase the robustness of the model to variations in seafood appearance. Additionally, the current framework does not account for dietary restrictions or allergies, which are critical considerations in food recommendation systems. Integrating allergen databases and dietary preference filters would make the framework more inclusive and practical for real-world use.

### 5.4. Comparison with Related Work

The integration of computer vision and intelligent agents for domain-specific recommendation represents an emerging frontier in agricultural and food informatics. The AGREE-YOLO framework proposed in this study is distinguished from existing approaches because of its holistic, agent-oriented pipeline that seamlessly couples visual ingredient detection with culturally contextualized LLM reasoning. In contrast, in prior research, these components have often been addressed in isolation or with limited cross-modal integration.

Traditional food recommendation systems predominantly rely on collaborative filtering or content-based textual analysis and lack the capacity to process raw visual inputs or adapt to diverse culinary contexts [34]. In recent works, deep learning has been used for food recognition, with models such as Mask R-CNN and Faster R-CNN being applied for ingredient identification [35,36]. However, these systems typically function as standalone detection modules and do not incorporate the detected ingredients into a structured, queryable knowledge base for downstream agent-based reasoning. Furthermore, although large language models (LLMs) have been leveraged for recipe generation, they primarily process textual prompts and lack integration with real-time visual detection systems, which limits their applicability in scenarios that involve physical food products [37,38].

In the specific domain of seafood, challenges such as morphological diversity and underwater imaging artifacts have been noted [12,39]. Although YOLO-series models have demonstrated state-of-the-art real-time detection capabilities [40,41], their application has been largely confined to pure detection tasks. Few studies have explored embedding these detection results within a relational database to facilitate dynamic, agent-driven retrieval and reasoning, which is a core innovation of the AGREE framework.

Concurrently, research on agricultural remote sensing has made significant progress in object extraction and segmentation and offers parallel insights. For instance, in recent work, the segment anything model (SAM) has been integrated with adaptive refinement strategies for precise cropland parcel extraction from satellite imagery, thus highlighting the importance of postprocessing modules for handling overlap and ensuring spatial continuity [42]. Similarly, multitask learning frameworks such as 3D reconstruction and the Swin transformer have been developed to jointly learn parcel features, boundaries, and spatial relationships, thereby improving geometric accuracy in complex landscapes [43,44]. These advancements underscore a broader trend of enhancing foundation models with task-specific, structured refinement mechanisms. However, these approaches focus on geographic object delineation and lack the agent-mediated, cross-cultural reasoning layer central to gastronomic recommendation.

The proposed AGREE-YOLO framework bridges these distinct research strands. It advances beyond mere detection by implementing a structured MySQL data layer, which is similar to the traceable geospatial data management approach used in precision agriculture [34,45,46]. Its ChatFlow pipeline and agent architecture in Dify introduce a novel paradigm for converting visual detections into actionable culinary intelligence, which represents a step beyond the static analysis performed in most computer vision or LLM-only systems. By unifying high-precision YOLO-based detection, structured database intermediation, and LLM-driven agent reasoning within a single pipeline, this work offers a more integrated and functionally complete solution for intelligent, culturally adaptive gastronomy than existing isolated or bimodal systems do.

Comparisons with state-of-the-art multimodal and agent-based food recommendation systems are systematically conducted to highlight methodological advancements [32]. The proposed AGREE-YOLO framework is quantitatively contrasted with two recent representative paradigms: the multimodal recipe recommendation system with multi-agent LLM reasoning and the closed-loop multi-agent nutrition management system driven by large language models [15]. Superior performance is demonstrated in cross-cultural adaptability, visual–textual integration, and end-to-end seafood-to-recipe automation, as neither competing system unifies YOLO-based detection, structured database querying, and culturally adaptive agent generation within a single pipeline.

Further comparative analyses are conducted against three recently published multimodal and agent-enhanced food recommendation systems published between 2024 and 2026. A vision–language and retrieval-augmented recipe recommendation framework named Pic2Plate is reported to rely on general visual ingredient extraction but lacks structured database storage and dedicated cross-cultural culinary agents [47]. Swin Transformer-based fine-grained food image recognition is adopted as a benchmark method [44]. While this method achieves high accuracy in visual identification, it is strictly unimodal and does not incorporate downstream recipe generation or cultural adaptation. Morales-Garzón et al. developed Adaptafood, an intelligent system for adapting recipes to specialized diets and healthy lifestyles using heterogeneous graph-based link prediction [48]. While this system excels in personalized dietary constraint handling, it lacks visual ingredient recognition capabilities and operates exclusively on textual and nutritional inputs without an agent-based reasoning layer for cultural customization. None of these three state-of-the-art systems support the unified pipeline of YOLO-driven seafood detection, structured MySQL querying, and culturally adaptive recipe generation, which confirms the unique advantages of the AGREE-YOLO framework in terms of technical integrity and gastronomic applicability.

As presented in Table 4, the proposed AGREE-YOLO framework outperformed all evaluated systems across the four quantitative metrics. The specific explanation is as follows:

“Cross-cultural adaptability” is defined as the degree to which the generated recipes align with region-specific culinary traditions, operationalized via the Conditional Country Selection (CCS) agent that selects target culinary frameworks (e.g., American, Japanese, Chinese) prior to recipe generation.

“Visual–textual integration fidelity” is defined as the accuracy of mapping YOLO-detected seafood instances to corresponding recipe components, measured at 89.5% for the proposed framework.

“Automation completeness” is defined as the extent to which a framework achieves full pipeline integration from raw seafood imagery to culturally adapted recipe and dish image outputs, with AGREE-YOLO scoring 100% as the only system achieving end-to-end integration.

For cross-cultural adaptability, AGREE-YOLO achieved a score of 92.7%, substantially higher than Adaptafood (45.8%), KERL (31.2%), and Pic2Plate (23.5%), a superiority attributed to the conditional country selection (CCS) agent operating within the Dify environment, which explicitly retrieves region-specific culinary knowledge prior to recipe generation. Regarding end-to-end automation completeness, AGREE-YOLO was the only framework achieving full pipeline integration (100%), representing a complete transformation from raw seafood imagery to culturally adapted recipe and dish image outputs, whereas Adaptafood (60%), Pic2Plate (40%), and KERL (20%) exhibited only partial integration due to missing structured data layers, cross-cultural modules, or visual detection capabilities. For visual–textual integration fidelity, AGREE-YOLO again led with 89.5%, reflecting accurate mapping from YOLO-detected instances to recipe components, compared with Pic2Plate (68.0%, limited by general-purpose detection on seafood targets), Adaptafood (52.0%, constrained by image-captioning extraction requiring complete dish images), and KERL (0%, lacking visual input). Finally, for database query accuracy among systems supporting structured retrieval, AGREE-YOLO achieved 94.3% accuracy in converting natural language prompts into valid SQL queries, notably exceeding KERL’s 56.7%, which was limited by entity extraction without explicit database schema awareness.

Objective quantitative metrics are strictly separated from qualitative descriptive analyses in all comparative statements. Measured performance values, including cross-cultural adaptability (92.7%), visual–textual integration fidelity (89.5%), end-to-end automation completeness (100%), and database query accuracy (94.3%), are reported as experimentally derived results. Qualitative interpretations regarding framework innovation, structural advantages, and methodological superiority are clearly labeled as descriptive assessments and distinguished from quantifiable outcomes. All comparative claims are anchored to measured numerical data rather than descriptive assertions alone.

## 6. Conclusions and Future Work

### 6.1. Conclusions

In this study, the AGREE-YOLO framework was successfully developed and validated as an integrated, agent-oriented system that connects real-time seafood recognition with culturally adaptive gastronomic recommendation. A lightweight YOLOv13 variant that was enhanced with GSConv modules and the Wise-IoU (WIoU) loss was implemented to achieve high-precision detection of three representative seafood ingredients—holothurians, echini, and scallops—under challenging underwater imaging conditions. The precision of the optimized model was 91.2%, and the recall was 87.3%, thus outperforming the baseline YOLOv13n by 3.9% and 4.2%, respectively, while maintaining a compact architecture with only 2.35 million parameters and an inference time of 2.0 ms. Detection results were systematically stored in a structured MySQL database with confidence thresholds enforced (≥0.83), thereby ensuring data reliability for downstream processing. Within the Dify environment, a novel ChatFlow pipeline was engineered to enable natural-language-based querying of the database, thereby facilitating seamless integration with LLM-driven agents. These agents dynamically generated culturally contextualized recipes—such as Creamy Lemon Garlic Scallops (American), Uni Gunkan Maki (Japanese), and Braised Sea Cucumber with Mushrooms (Chinese)—paired with visually coherent dish images. Operational validation confirmed the ability of the framework to translate raw visual inputs into personalized, cross-cultural culinary recommendations that account for both regional practices and psychological dimensions of user satisfaction. Collectively, the AGREE-YOLO framework provides a viable pathway for unifying computer vision, structured data management, and agent-mediated reasoning in intelligent food systems, thus offering a foundation for future applications in sustainable seafood utilization, culinary education, and culturally responsive AI-driven gastronomy.

### 6.2. Future Directions

In future work, attention will be focused on addressing the limitations of the current framework and expanding its capabilities. First, the seafood detection model will be extended to include a wider range of ingredients, with dataset expansion and transfer learning techniques used to maintain detection accuracy. Second, the cultural adaptation module will be enhanced to support additional culinary traditions and sub-cuisines, with the integration of cultural cuisine databases and expert knowledge to ensure authenticity. Third, user feedback mechanisms will be incorporated to enable personalized recommendations on the basis of individual preferences, dietary restrictions, and psychological responses.

The VLM-driven agent system is optimized for efficiency and scalability. Optimizations include gastronomic dataset fine-tuning, specialized agent design, and edge computing integration. The framework will also be extended to support multimodal inputs (e.g., text, image, and voice inputs) and outputs (e.g., video recipes and interactive cooking guides), thereby increasing its accessibility and user-friendliness.

Finally, the AGREE framework will be validated in real-world settings, such as seafood markets, restaurants, and culinary education platforms, to assess its practical utility and gather user feedback. This will enable iterative refinement of the framework and ensure its alignment with real-world needs. By addressing these directions, the AGREE-YOLO framework has the potential to become a leading tool in intelligent gastronomy that connects deep learning, cultural diversity, and user-centric satisfaction to provide a more engaging and inclusive culinary experience.

## Figures and Tables

**Table 1:** Database schema for seafood detection records.

Column Name	Data Type	Constraints	Purpose
Detection_ID	INT	PRIMARY KEY, AUTO_INCREMENT	Unique index for each detection event
Yolo_Confidence_Score	DECIMAL (3.2)	CHECK (yolo_confidence_score ≥ 0.83)	Ensures high-reliability detection data
Detection_Timestamp	DATETIME	NOT NULL	Timestamp for detection traceability and synchronization
Image_Source_ID	VARCHAR (80)	UNIQUE, NOT NULL	Enables traceability to original imagery
Nutritional_Ingredient	VARCHAR (60)	NOT NULL, ENUM (‘Holothurian’, ‘Echinus’, ‘Scallop’)	Standardized ingredient classification

**Table 2:** Hyperparameters for enhanced lightweight YOLOv13n training.

Hyperparameter	Value
batch size	16
learning rate	0.01
epochs	300
imgsz	640
mosaic	1.0
mixup	0.0
copy_paste	0.1

**Table 3:** Comparison of the performance of object detection models for seafood ingredient recognition across key operational metrics.

Model	Inference Time (ms)	P (%)	R (%)	Params (M)	GFLOPS
SSD	7.38	0.613	0.546	14.12	36.4
Faster R-CNN	46.3	0.764	0.685	44.18	203.7
YOLOv8n	1.2	0.859	0.823	2.58	6.6
YOLOv11n	1.3	0.867	0.835	2.51	6.4
YOLOv13n	2.2	0.873	0.831	2.45	6.2
YOLOv13n-GSConv-WIoU (Ours)	2.0	0.912	0.873	2.35	6.1

**Table 4:** Quantitative comparison of AGREE-YOLO against state-of-the-art multimodal and agent-based food recommendation systems.

System	Visual Input	Seafood Detection Precision (%)	Cross-Cultural Adaptability (%)	End-to-End Automation (%)	Visual–Textual Integration (%)	Database Query Accuracy (%)	Image Generation
Pic2Plate [47]	Yes	77 *	23.5	40	68.0	N/A ^†^	No
KERL [32]	No	N/A	31.2	20	0	56.7	No
Adaptafood [48]	Yes	0 ^‡^	45.8	60	52	N/A ^†^	No
AGREE-YOLO (Ours)	Yes	91.2 ^‡^	92.7	100	89.5	94.3	Yes

Note: * Pic2Plate reported 83.0% precision on general food images; the 77.0% value represents performance on the seafood-specific test set used in this study; ^†^ Systems without structured database query capability; ^‡^ Adaptafood requires recipe text or image inputs containing multiple ingredients; it does not perform single-ingredient seafood detection.

## Data Availability

The original contributions presented in this study are included in the article. Further inquiries can be directed to the corresponding author.

## References

[1] De Cock A., Forio M.A.E., Hansen H.H., Jacxsens L., Lachat C., Goethals P. (2025). Integrated control of seafood safety and quality beyond farm-to-fork: AI-related opportunities and challenges. Trends Food Sci. Technol..

[2] Xu M.Y., Fang D.L., Kimatu B.M., Lyu L., Wu W.L., Cao F.L., Li W.L. (2024). Recent advances in anthocyanin-based films and its application in sustainable intelligent food packaging: A review. Food Control.

[3] Zhu J.D., He W.W., Weng W.D., Zhang T., Mao Y.Z., Yuan X.T., Ma P.Z., Mao G.J. (2022). An Embedding Skeleton for Fish Detection and Marine Organisms Recognition. Symmetry.

[4] Taylor B., Ofori K.F., Parsaeimehr A., Evrendilek G.A., Attarwala T., Ozbay G. (2025). Exploring the Complexities of Seafood: From Benefits to Contaminants. Foods.

[5] Mahbubi A., Fatoni A., Iskandar S.P., Yunita E. (2025). Promoting local food into gastro-tourism at the Gunung Kubing Geosite of Belitong UNESCO global geopark. Int. J. Gastron. Food Sci..

[6] Ge C., Zhang G.J., Wang Y.J., Shao D.D., Song X.J., Wang Z.W. (2025). Research Status and Development Trends of Artificial Intelligence in Smart Agriculture. Agriculture.

[7] Wu H.Y., Jia B.W., Yuan X.M. (2026). LLM-led vision-spectral fusion: A zero-shot approach to temporal fruit image classification. Neural Netw..

[8] Li H.L., Li J., Wei H.B., Liu Z., Zhan Z.F., Ren Q.L. (2024). Slim-neck by GSConv: A lightweight-design for real-time detector architectures. J. Real-Time Image Process..

[9] Wang J.L., Qin C.C., Hou B.B., Yuan Y., Zhang Y.K., Feng W.F. (2024). LCGSC-YOLO: A lightweight apple leaf diseases detection method based on LCNet and GSConv module under YOLO framework. Front. Plant Sci..

[10] Su B., Zhu Y.Y., Lin Y.F. (2025). Pest-YOLOv8: Enhanced detection for small and complex agricultural pests using triple attention and wise-IoU. J. Plant Dis. Prot..

[11] Tang Y.S., Zhang Y., Xiao J.R., Cao Y., Yu Z.J. (2024). An Enhanced Shuffle Attention with Context Decoupling Head with Wise IoU Loss for SAR Ship Detection. Remote Sens..

[12] Fu T.Q., Hu Q., Zhao J.W., Jiang G.Y., Shan L.H., Rong Y. (2025). Underwater target detection and recognition based on cross-modal fusion of flow and electric information. Measurement.

[13] Joshi A., Pandey N., Diwakar M., Singh P., Shankar A., Alqahtani F. (2025). WBD-YOLO-AM: YOLOv8 with attention module and IoT-based wild boar detection and deterrence system for safeguarding small millets. Turk. J. Agric. For..

[14] Zhang W.X., Shi X.W., Jiang M.L., Zhang A., Zeng L.G., Al-qaness M.A.A. (2025). Improved you only look once for weed detection in soybean field under complex background. Eng. Appl. Artif. Intell..

[15] Dong C.L., Ying H.C., Hu R.J., Xu Y.Y., Chen J.T., Zhuang F.Z., Wu J. (2025). A Progressively-Passing-Then-Disentangling Approach to Recipe Recommendation. IEEE Trans. Multimed..

[16] Foster J., Brintrup A. (2025). Aiding food security and sustainability efforts through graph neural network-based consumer food ingredient detection and substitution. Sci. Rep..

[17] Vats M., Flinders B., Visvikis T., Dawid C., Hofmann T.F., Cuypers E., Heeren R.M.A. (2024). Mass Spectrometry Imaging for Spatial Ingredient Classification in Plant-Based Food. J. Am. Soc. Mass Spectrom..

[18] Enriquez J.P., Archila-Godinez J.C. (2022). Social and cultural influences on food choices: A review. Crit. Rev. Food Sci. Nutr..

[19] Hu Y., Zhang X.M., Fang Y.K., Gao Z.F. (2025). The influence of multicultural experience on attitudes towards new foods in the U.S. Appetite.

[20] Khanna S.K. (2021). Cultural Influences on Food: Dietary and Health Implications. Ecol. Food Nutr..

[21] Krzyzewska A. (2025). AI Advice for Amateur Food Production: Assessing Sustainability of LLM Recommendations. Sustainability.

[22] Mamun A., Arefeen A., Racette S.B., Sears D.D., Whisner C.M., Buman M.P., Ghasemzadeh H. (2025). LLM-Powered Prediction of Hyperglycemia and Discovery of Behavioral Treatment Pathways from Wearables and Diet. Sensors.

[23] Wang X.L., Min W.Q., Sheng G.R., Song J.R., Yang Y.C., Yao T., Jiang S.Q. (2026). LLM-informed global-local contextualization for zero-shot food detection. Pattern Recognit..

[24] Nian F.D., Hu Y.J., Gu Y.H., Wu Z.Z., Yang S.M., Shu J.H. (2024). Ingredient-guided multi-modal interaction and refinement network for RGB-D food nutrition assessment. Digit. Signal Process..

[25] Qiao G.H., Nong L.M., Cheng C.Y., Shen Z.W., Zhu J.L., Li H. (2026). ProFood: Progressive RGB-D fusion network for food detection in complex diet scenes. J. Food Compos. Anal..

[26] Zhang E.S., Li A.Z., Zhang G.X., Lu W.H., Zhang Q.X., Chen L., Jiang L., Ju P., Qu F.L. (2025). A shikimic acid derived carbon dots (SACNDs-FITC) for multi-modal detection and removal of Hg^2+^: Probe design, sensing performance, and applications in food analysis. Spectrochim. Acta Part A-Mol. Biomol. Spectrosc..

[27] Öttl A., Termansen M. (2025). Agent-Based Modelling of food systems: A scoping review on incorporation of behavioural insights. Environ. Model. Softw..

[28] Wang Y.K., Yang Y., Slanzi C.M., Li X.L., Ojeda A., Paro F., Deblais L., Yakubu H., Hassen B.M., Game H. (2025). Quantitative multi-pathway assessment of exposure to *Escherichia coli* for infants in Rural Ethiopia. PLoS Neglected Trop. Dis..

[29] Li J.X., Yan Z.P. (2026). EAMSF-DETR: Edge-aware multi-scale feature fusion network based on DETR for underwater object detection. Opt. Laser Technol..

[30] Hu Z.Y., Chen Q. (2025). MOA-YOLO: An Accurate, Real-Time, and Lightweight YOLOv10-Based Algorithm for Deep-Sea Fish Detection. IEEE Sens. J..

[31] Mou D., Wei Z.H., Ni L., Song N., Sun Y.W., Chu W.Z., Jin B.K. (2025). LLM-enhanced representation learning for graph collaborative filtering recommendation models. J. Intell. Inf. Syst..

[32] Kopitar L., Bedrac L., Strath L.J., Bian J., Stiglic G. (2025). Improving Personalized Meal Planning with Large Language Models: Identifying and Decomposing Compound Ingredients. Nutrients.

[33] Chen J., Er M.J. (2025). A hybrid architecture based on structured state space sequence model and convolutional neural network for real-time object detection. Eng. Appl. Artif. Intell..

[34] Ahmadian S., Rostami M., Jalali S.M.J., Oussalah M., Farrahi V. (2025). A healthy and reliable rating profile expansion approach to address data sparsity in food recommendation systems. Knowl. Inf. Syst..

[35] Cao Y.Y., Zhao Z.X., Huang Y., Lin X., Luo S.Y., Xiang B.R., Yang H.C. (2023). Case instance segmentation of small farmland based on Mask R-CNN of feature pyramid network with double attention mechanism in high resolution satellite images. Comput. Electron. Agric..

[36] Feng J.H., Zhao X.R., Zhu T.Y., Li T., Qiu Z.C., Li Z.W. (2023). Detection mature bud for daylily based on Faster R-CNN integrated with CBAM. IEEE Access.

[37] Ma P.H., Tsai S.W., He Y.Y., Jia X.X., Zhen D.Y., Yu N., Wang Q., Ahuja J.K.C., Wei C. (2024). Large language models in food science: Innovations, applications, and future. Trends Food Sci. Technol..

[38] Rezayi S., Liu Z.L., Wu Z.H., Dhakal C., Ge B., Dai H.X., Mai G.C., Liu N.H., Zhen C., Liu T.M. (2025). Exploring New Frontiers in Agricultural NLP: Investigating the Potential of Large Language Models for Food Applications. IEEE Trans. Big Data.

[39] Chen H.Y., Li Y.Z., Xu P., Li J.Q., Noor A., Zhou X.Y., He W.C., Wang T.R., Mou Z.Y., Song L.G. (2025). Octopus-inspired soft gripper with embedded triboelectric tactile sensor for underwater target recognition and grasp. Nano Energy.

[40] Dong K., Li D.Y., Zhang J.J., Zhao X.L., Dong L.J., Zhang P. (2025). RV-YOLO: Real-time object detection algorithm for rail transit platform scenarios. J. Real-Time Image Process..

[41] Su Z.B., Zhao K.Q., Fan Z.H., Guo X.H. (2025). RTPV-YOLO: Real-Time Photovoltaic Detection With UAV-Based Thermal and RGB Imaging. IEEE Trans. Aerosp. Electron. Syst..

[42] Li H.B., Zhu J.Y., Mao X., Hao X.L., Li S.Y., Yu Q.Y., Shi Y., Qian J.P. (2026). Achieving precise cropland parcel extraction from remote sensing images through integration of segment anything model and adaptive mask refinement. Comput. Electron. Agric..

[43] Chen J.L., Cui Q.W., Ye Y. (2025). 3D reconstruction and landscape restoration of garden landscapes: An innovative approach combining deep features and graph structures. Front. Environ. Sci..

[44] Xiao Z.Y., Diao G., Deng Z.H. (2024). Fine grained food image recognition based on swin transformer. J. Food Eng..

[45] Kostic M., Sarac V., Narandzic T., Kovacevic D.B. (2026). Digital and Green Technological Drivers of Transformation in the Agri-Food Sector. Foods.

[46] Benyezza H., Bouhedda M., Kara R., Rebouh S. (2023). Smart platform based on IoT and WSN for monitoring and control of a greenhouse in the context of precision agriculture. Internet Things.

[47] Soekamto Y.S., Lim A., Limanjaya L.C., Purwanto Y.K., Lee S.H., Kang D.K. (2025). Pic2Plate: A Vision-Language and Retrieval-Augmented Framework for Personalized Recipe Recommendations. Sensors.

[48] Morales-Garzón A., Gutiérrez-Batista K., Martin-Bautista M.J. (2025). Adaptafood: An intelligent system to adapt recipes to specialised diets and healthy lifestyles. Multimed. Syst..

